# The complexity of anatomical systems

**DOI:** 10.1186/1742-4682-2-26

**Published:** 2005-07-19

**Authors:** Fabio Grizzi, Maurizio Chiriva-Internati

**Affiliations:** 1 Scientific Direction, Istituto Clinico Humanitas, IRCCS, Via Manzoni 56, 20089 Rozzano, Milan, Italy; 2 Michele Rodriguez Foundation, Scientific Institute for Quantitative Measures in Medicine, Via Ludovico Di Breme 79, 20100 Milan, Italy; 3 Department of Microbiology & Immunology, Texas Tech University Health Sciences Center and Southwest Cancer Treatment and Research Center, 79430 Lubbock, Texas, USA

## Abstract

**Background:**

The conception of *anatomical entities *as a hierarchy of infinitely graduated forms and the increase in the number of observed anatomical sub-entities and structural variables has generated a growing *complexity*, thus highlighting new properties of organised biological matter.

**Results:**

(1) Complexity is so pervasive in the anatomical world that it has come to be considered as a primary characteristic of anatomical systems. (2) Anatomical entities, when viewed at microscopic as well as macroscopic level of observation, show a different degree of complexity. (3) Complexity can reside in the *structure *of the anatomical system (having many diverse parts with varying interactions or an intricate architecture) or in its *behaviour*. Often complexity in structure and behaviour go together. (4) Complex systems admit many descriptions (ways of looking at the system) each of which is only partially true. Each way of looking at a complex system requires its own description, its own mode of analysis and its own breaking down of the system in different parts; (5) Almost all the anatomical entities display hierarchical forms: their component structures at different spatial scales or their process at different time scales are related to each other.

**Conclusion:**

The need to find a new way of observing and measuring anatomical entities, and objectively quantifying their different structural changes, prompted us to investigate the non-Euclidean geometries and the theories of complexity, and to apply their concepts to human anatomy. This attempt has led us to reflect upon the complex significance of the shape of an observed anatomical entity. Its changes have been defined in relation to variations in its *status*: from a normal (*i.e. *natural) to a pathological or altered state introducing the concepts of *kinematics *and *dynamics *of anatomical forms, *speed *of their changes, and that of *scale *of their observation.

## Background

Since the early 1950s, the concept of *spatial conformation *in general inorganic, organic and particularly biological chemistry has assumed a fundamental role in the study of the various properties of biological macromolecules (nucleic acids, proteins, carbohydrates, lipids) [1]. Because of the technologies of three-dimensional analysis, this concept is currently used in modern biology. The biological polymers that have been most widely studied in structural and functional terms are proteins and nucleic acids (DNA and RNA) [2-5].

It is now well established that the information needed to determine the three-dimensional structure of a protein is entirely contained in its linear amino acid sequence. It is likewise known that abrupt changes in environmental conditions (pH, temperature, pressure) may reversibly or irreversibly alter the tri-dimensional structure of a biological macromolecule, and thus change its specific function [6]. However, *conformational change *is a still widely discussed concept. The definition of the *spatial conformation *of either a microscopic or a macroscopic *anatomical structure *(sub-cellular entity, cell, tissue, organ, apparatus, organism), and the definition of a change or *modification *in its *shape*, are still unresolved problems, much debated by contemporary morphologists [7-12].

In its general sense, the term *structure *denotes the property resulting from the configurations of the parts that form a Whole and their reciprocal relationships to each other and to the Whole itself. On the basis of this definition, two properties of all anatomical systems made up of *organised biological matter *can be highlighted:

a. every anatomical structure is capable of expressing a particular function in a particular context;

b. the different configurations and functions of an anatomical entity emerge from structures organised in overlapping hierarchical levels.

The term 'organised biological matter' denotes anything that (1) has its own *shape *and *dimension*, i.e. space-filling property, and (2) can reproduce or replicate itself in such a way as to give rise to 'entities' that are similar in shape, dimension and functional properties to their progenitors.

It is well known that human cells differ in their shapes, dimensions and sizes. All cells making up an adult organism derive from a single progenitor cell, from which arises an enormous number of cells with different shapes, dimensions, sizes, chemical compositions and physiological characteristics in a complex and dynamic process known as *cell differentiation *[1,13].

**Figure 1:**
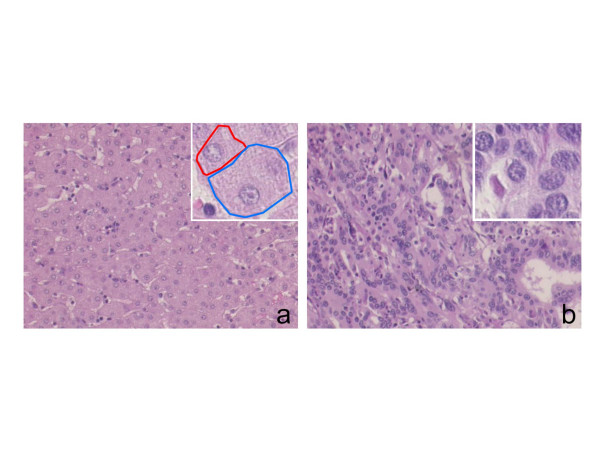
Intra-cellular and/or extra-cellular stimuli determine the shape of an animal cell. In many cases intricate relationships between sub-cellular entities, such as the cytoskeleton, and environmental variables influence the cell's *shape*, *dimension *and *size*. Liver parenchymal cells, called *hepatocytes*, are roughly polyhedral *in situ (a) *but when they are dissociated and immersed in a culture medium gradually take on a spherical shape. Tumoral liver cells may drastically change their morphological characteristics, as result of a high number of variables that influence the global behaviour of the cell *(b).*

Certain cells have specific, particular and consequently invariable characteristic shapes, regardless of whether they are isolated or grouped to form more complex anatomical entities known as *tissues *(Figure 1). However, other cells are subject to *conformational changes *that depend particularly on the mechanical action exerted by their environment, the compression induced by contiguous cells, and either the complicated relationships between the cells and the *extra-cellular matrix *involved in the creation of tissue, or the surface tension of the biological fluid in which the cells are immersed [11,12].

Liver parenchymal cells (*hepatocytes*) are roughly polyhedral *in situ *but, when they are dissociated and immersed in a culture medium, gradually take on a spherical shape (Figure 1) [14,15]. It has been widely demonstrated that grouped cells respect the laws of *cytomorphogenesis *(morphogenetic cell development) by maximally exploiting the space available to them [7]. The *variability *or *constancy *of cell shape also depends on the physical support provided by the internal *cytoskeleton *[16-20].

The fact that all living organisms can be classified on the basis of their appearance is an important indication that each has a *specific form *(i.e. one that is retained by every example of the same species). The morphological criterion is therefore of considerable importance in identifying and taxonomically classifying living organisms.

Our aim here is to give meaning to the complex forms characterising anatomical entities in a similar way to that offered by spatial conformation in the chemical sciences. This attempt has led us to reflect upon and discuss the complex significance of the shape of an observed anatomical entity. Its changes have been defined in relation to variations in its status: from a normal (*i.e. *natural) to a pathological or altered state, introducing the concepts of *kinematics *and *dynamics *of anatomical forms, that of *speed *of their changes, and that of *scale *of their observation.

## The complexity of living systems

Unlike an anatomical entity, and despite the fact that it has a unique shape, a *crystal *has no unequivocally defined size that can be used for classification; a small crystal of a given substance will always have the same general structure as a large crystal of the same type.

Any *fragment *of a crystal has the same physical and chemical characteristics as the whole crystal, but this is not true of any fragment of a living organism because the chemical compositions and physical properties of the individual parts do not correspond with the composition of the Whole. Furthermore, the various components of a living system are characterised by the *integration *of precise functional criteria that form a Whole [21].

**Figure 2:**
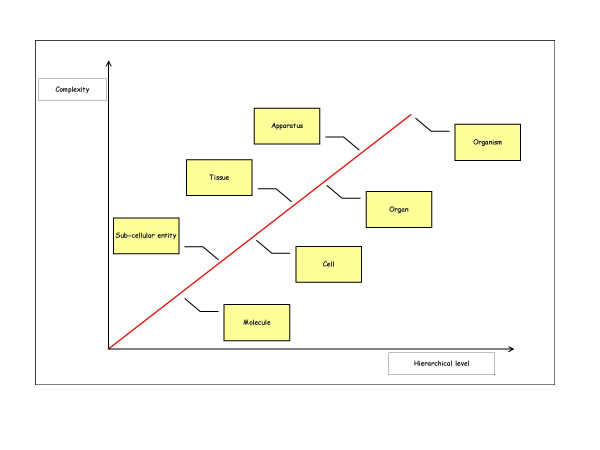
Human beings are complex hierarchical systems consisting of a number of hierarchical levels of anatomical organization (molecules, sub-cellular entities, cells, tissues, organs, apparatuses, and organism) that interrelate differently with each other to form networks of growing *complexity.*

Returning once again to crystals, their macroscopic structures can easily be predicted on the basis of their microscopic structures; they lack what are called *emergent properties*: *i.e. *those that strictly depend on the level of organisation of the material being observed (Figure 2).

The existence of different organisational levels governed by different laws was first indicated by *systemist biologists*, who stressed that a fundamental characteristic of the *structural organisation *of living organisms is their *hierarchical nature *(Figure 2). One of the pre-eminent characteristics of the entire living world is its tendency to form multi-level structures of "systems within systems", each of which forms a Whole in relation to its parts and is simultaneously part of a larger Whole.

*Systemism *was born in the first half of the twentieth century as a reaction to the previous *mechanistic movement *(also known as *reductionism*). It was based on an awareness that classical causal/deterministic schemata are not sufficient to explain the variety of interactions characterising living systems. Advances in the fields of cybernetics and biology led to the proposition of new interpretative models that were better suited to identifying and describing the *complexity of phenomena *that could no longer be seen as abstractly isolated entities divisible into parts or explicable in terms of temporal causality, but needed to be studied in terms of the dynamic interactions of their parts. The word *system *means "putting together". Systemic understanding literally means putting things in a context and establishing the nature of their relationships, and implies that the phenomena observed at each level of organisation (molecules, sub-cellular entities, cells, tissues, organs, apparatuses and organisms) have properties that do not apply lower or higher levels (Figure 2).

As we have already said, according to systemic thought, the essential properties of a living being belong to the Whole and not to its component parts. This led to the fundamental discovery that, contrary to the belief of René Descartes, biological systems cannot be understood by means of *reduction *[21-24]. The properties of the individual component parts can only be understood in the context of the wider Whole.

The biologist and epistemologist Ludwig von Bertalanffy provided the first theoretical construction of the complex organisation of living systems [25]. Like other organic biologists, he firmly believed that to understand biological phenomena, new modes of thought that went beyond the traditional methods of the physical sciences were required [26,27]. According to Bertalanffy, living beings should be considered as complex systems with specific activities to which the principles of the thermodynamics of "closed" systems studied by physicists do not apply. Unlike *closed systems *(in which a state of *equilibrium *is established), open systems remain in a *stationary state far from equilibrium *and are characterised by the *input *and *output *of *matter*, *energy *and *information *[28].

James Grier Miller first introduced the *Living System Theory *(LST) about how living systems 'work', how they maintain themselves and how they develop and change [29]. By definition, living systems are open, *self-organizing systems *that have the peculiar characteristics of life and interact with their environment. This takes place by means of information, matter and energy exchanges. The term *self-organization *defines an evolutionary process where the effect of the environment is minimal, *i.e. *where the generation of new, complex structures takes place fundamentally in and through the system itself [30,31]. In open systems, it is the continuous flow of matter and energy that allows the system to self-organize and to exchange *entropy *with the environment. Supported by a plethora of scientific data, LST asserts that all the great variety of living entities that evolution has generated are complexly structured open systems [32]. They maintain thermodynamically improbable energy states within their boundaries by continuous interactions with their environments [32-34].

LST indicates that living systems exist at eight levels of increasing complexity: *cells*, *organs*, *organisms*, *groups*, *organizations*, *communities*, *societies*, and *supranational systems *[29,32-34]. All living systems are organized into critical *subsystems*, each of which is a structure that performs an essential life process. A subsystem is thus identified by the process it carries out. LST is resulted an integrated approach to studying biological and social systems, the technology associated with them, and the ecological systems of which they are all parts [35,36].

Exploration of the phenomena of life at increasingly microscopic levels (*genome*) showed that the characteristics of all living systems are encoded in their *chromosomes *by means of a single chemical substance that has a universal transcription code [1]. In this sense, biological research became largely reductionist (*i.e. *increasingly involved in the analysis of molecular details). Like its seventeenth-century mechanistic predecessor, it produced an enormous amount of significant data concerning the precise structure of individual genes without knowing how these *communicate *and *cooperate *with each other in the development of an organism and its structural and functional modifications. Through continuing fundamental advances in molecular and cellular biology, molecular biologists discovered the basic *building bricks *of life, but this did not help them to understand the fundamental integrational processes of living beings [21-24]. As Sidney Brenner said: *"In one way, you could say all the genetic and biological work of the last sixty years could be considered a long interlude....We have come full circle – back to the problems left behind unsolved. How does a damaged organism regenerate with exactly the same structure it had before? How does the egg form the organism? ....In the next twenty-five years, we are going to have to teach biologists another language....I do not know yet what its name is; nobody does... ...It is probably wrong to believe that all logic lies at molecular level. It may be that we will need to go beyond the mechanisms of a clock" *[29].

In fact, a new language has emerged over the past few years that makes it possible to interpret and understand living organisms as highly integrated systems [26,37-46]. Based on the concept of the *complexity *of the living, this language has given rise to several branches of study concerning the structure and organization of living organisms (such as the fractal geometry of Benoit Mandelbrot and other non-Euclidean geometries [47]) and the biological phenomena that take place within them (such as the Theory of Dynamic Systems, the Catastrophe Theory of René Thom, and the Chaos Theory [48-52]).

## The kinematics and dynamics of anatomical forms

**Figure 3:**
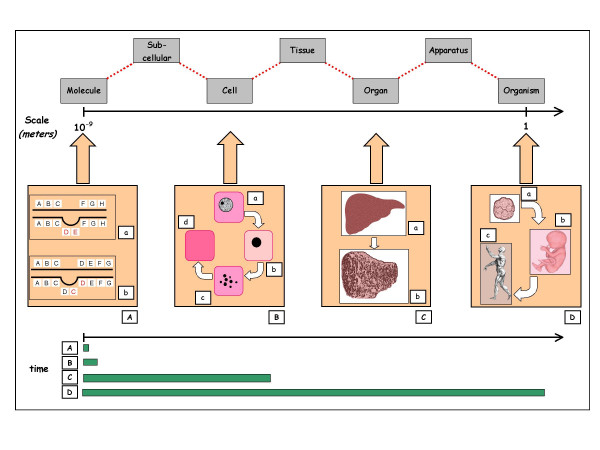
Complex dynamical changes in humans at different level of spatial organization. **A. **Examples of chromosomal alterations (mutations): *a) deletion *of a tract of DNA; *b) duplication *of a tract of DNA sequence. **B. **The progressive changes occurring in the nucleus and cytoplasm that accompany the death of a cell. *a) *Normal cell; *b) *The nucleus becomes contracted and stains intensely. The cytoplasm is pinker, showing that it binds *eosin *(a common histochemical stain) more avidly. *c) *The nucleus disintegrates, appearing as a more or less central area of dispersed chromatin. This phase is called karyorrhexis. *d) *All nuclear material has now disappeared (kariolysis) and the cytoplasm stains an intense red colour. **C. **The final appearance of the liver *(a) *when it assumes the state of cirrhosis *(b)*. Cirrhosis is the final stage of several pathogenic mechanisms operating either alone or in concert to produce a liver diffusely involved by fibrosis (abnormal extra-cellular matrix deposition) and the formation of structurally abnormal parenchymal nodules. **D. **Human life: from the embryonic stage of morula *(a)*, through that of foetus *(b)*, to the adult being *(c)*. The times elapsing in the variousdynamical processes exemplified (A-D) are very different *(simplified by green bars)*, ranging from *nanoseconds *to *years*. It is interesting to highlight the *inverse relationship *between the *level of anatomical complexity *and *timescale.*

It would therefore be desirable to introduce the concept of the *complexity of form *into the anatomical sciences and encourage awareness that an anatomical structure observed at sub-microscopic level is governed by different laws when it is observed at microscopic or macroscopic level (Figure 3).

One of the fundamental problems facing the human mind is that of the *succession of forms*, introduced by René Thom in his book "Stabilité Structurelle et Morphogenèse. Essai d'une théorie générale des modèles", first published in 1972 [48]. Whatever the ultimate nature of reality may be, it is undeniable that our Universe contains a variety of natural objects and living beings. These things and beings are forms: *i.e. *structures equipped with a certain morphological and functional stability that occupy a certain portion of space and last a certain length of time. It is a commonplace that the Universe is an incessant *birth*, *development*, and *destruction *of forms [48].

**Figure 4:**
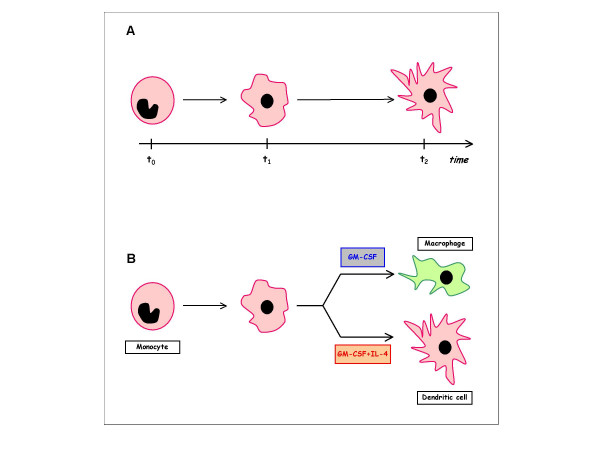
Kinematics and dynamics of human dendritic cells and macrophage differentiation *in vitro*. Cultured in vitro, monocytes may change their shape, dimension and size when opportunely stimulated by specific growth factors. Kinematics studies these changes without considering the nature of the entities to which they belong or what causes the changes *(A)*. Cultivation *in vitro *with Granulocyte Macrophage-Colony Stimulating Factor (GM-CSF) alone or with Interleukin-4 (IL-4) selectively determines differentiation into macrophages or dendritic cells *(B)*. In this case the study of the temporal transformations of primary monocytes in relation to the causes determining the changes, is defined as dynamics of the anatomical forms.

The *succession of anatomical forms *thus brings us to define:

a. The *kinematics of anatomical forms*, which studies *temporal transformations *of an anatomical form without considering the nature of the entities to which it belongs or what causes changes (Figure 4a). When an anatomical form changes, one or more of its qualities is modified in comparison with analogous anatomical forms that are considered unchanged: *e.g. *a cell can change its shape or one of its associated qualities in a tissue in which other cells remain unchanged. The set of unchanged anatomical forms is called the *reference system*. A cell can therefore be said to be in a state of *morphological stability *or a *phase of modification *in relation to a particular reference system, depending on whether its shape remains the same or varies over time in comparison with the other cells in the system (*i.e. *the tissue).

b. The *dynamics of anatomical form*, which studies the *temporal transformations *of an anatomical form in relation to the causes of the changes. An anatomical form in a state of morphological stability tends to preserve its shape in the surrounding space. However, if we apply any (internal or external) *factor ****u***, it abandons this state of 'rest' and enters a *phase of modification *(Figure 4b). This factor, which can be considered a true *physical force*, may act on the elements determining the *shape *of the system (*e.g. *in the cell system: the plasmalemma or cytoskeleton) and/or those determining its function or its *internal points *(*e.g. *the nucleus, mitochondria, and the smooth and rough endoplasmic reticulum) [53]. The change in shape can be considered as a *non-linear dynamic system *that advances through *states *that are *qualitatively *different (Figure 4). The word 'state' denotes the *pattern configuration *of a system at a particular instant, which is specified by a large number of dynamic variables. A dynamic system can be characterised by a set of different states or possible pattern configurations (**x**) and a number of *transitions *or steps (x) from one state to another during a certain time interval (*t*). When the transitions are caused by a generating element (**u**), the temporal behaviour of the system can be described by the general equation:

**x **= *f *(**x**, **u**, *t*)

where *f *is a *non-linear function *and the dot denotes a differentiation with respect to time (*t*).

c. The *speed of change *is the time necessary for a change in shape to occur or for the development of a perceptible difference between the modified entity and its unchanged reference system. In quantitative terms, it means the rapidity of the transformation of the anatomical form. However, the parameter *time *depends on a large number of variables that are interconnected in a multitude of ways and in a non-linear manner [53]. This makes it extremely difficult to predict the exact time interval between two successive states. Although conformational changes are a *continuum*, differentiation into successive states is commonly based on differences in *shape*, *dimensions *or *functional activity *(Figure 4).

Modelling the complexity of living beings should take into account the 10–12 order-of-magnitude span of timescales for events in biological systems, whether molecular (*ion channel gating*: 10^-6 ^seconds), cellular (*mitosis*: 10^2^-10^3 ^seconds), or physiological (*cancer progression*, *ageing*: 10^8 ^seconds).

d. The *scale of observation*, by which is meant the level at which the interrelated parts of a complex structure is being studied.

It must be emphasised that observed morphological patterns can often be conceptualised as *macro-scale *manifestations of *micro-scale *processes. However, observed patterns or system states are created or influenced by multiple processes and controls. Furthermore, those multiple processes operate at multiple *spatial *and *temporal *scales, both larger and smaller than the scale of observation.

It is also necessary to highlight that there is no one 'true' value for a measurement [52]. The measured value of any property of a biological object depends on the characteristics of the object. When these characteristics depend on the resolution of measurement, then the value measured depends on the measurement resolution. This dependence is called the *scaling *relationship [47]. *Self-similarity *specifies how the characteristics of an object depend on the resolution and hence determines how the value measured for a property depends on the resolution [47,52].

## Conclusive key points

One of the basic problems in evaluating complex living forms and their changes is how to analyse them quantitatively. Although mathematical thought has not had the same impact on biology and medicine as on physics, the mathematician George Boole pointed out that the *structure of living matter is subject to numerical relationships *in all of its parts, and that all its dynamic actions are measurable and connected by defined numerical relationships. Boole saw human thought in mathematical terms and, given its nature, mathematics holds a fundamental place in human knowledge.

The origins of the interest of mankind in the *mathematics of form *go back to ancient times, when it coincided with the manifestation of specific practical needs and, more generally, the need to describe and represent the surrounding world. The use of geometry to describe and understand *reality *is essential insofar as it makes it possible to reconstruct the inherent rational order of things. According to Pythagoras, real knowledge was necessarily mathematical. This idea continued until the early years of the seventeenth century, when Galileo re-proposed the observations made by Pythagoras, with no substantial modification, by affirming that the Universe is written in the language of mathematics, whose letters are triangles, circles and other geometric figures.

However, during the first half of the twentieth century, it was discovered that the geometric language of Euclid is not the only possible means of making axiomatic formulations, but that other geometries exist that are as self-consistent as classical geometry. This led to the flourishing of new geometrical languages capable of describing new spatial imaginations in rigorous terms. While successive generations of mathematicians were elaborating a large number of new non-Euclidean geometries, the beginning of the twentieth century saw the discovery of mathematical objects that seemed at first sight to be little more than curiosities devoid of practical interest (to the extent that they were even called 'pathological'). However, in the mid-1970s, the mathematician Benoit Mandelbrot gave them new dignity by defining them as "fractal objects" and introducing with them a new language called "fractal geometry".

Fractal geometry moves in a different developmental direction from the non-Euclidean geometries. Whereas the latter are based on the collocation of familiar objects in spaces other than Euclidean space, fractal geometry stresses the nature of geometric objects regardless of the ambient space. The novelty of fractal objects lies in their infinite morphological complexity, which contrasts with the harmony and simplicity of Euclidean forms but matches the variety and wealth of *complex natural forms*.

In conclusion, we can highlight that the following points:

*a) *Complexity is so pervasive in the anatomical world that it has come to be considered a basic characteristic of anatomical systems.

*b) *Anatomical entities, viewed at *microscopic *and *macroscopic *level of observation, show different *degrees of complexity*.

*c) *Complexity can reside in the *structure *of the system (having many diverse parts with varying interactions or an intricate architecture) or in its *behaviour*. Often, complexity in structure and behaviour go together.

*d) *A complex system admits many descriptions (ways of looking at the system), each of which is only partially true. Each way of looking at a complex system requires its own description, its own mode of analysis and its own breakdown of the system into different parts;

*e) *Almost all anatomical entities display *hierarchical *forms: their component structures at different spatial scales, or their process at different time scales, are related to each other.

Application of these concepts promises to be useful for analyzing and modelling the real significance of the shape, dimension and size of an observed anatomical system at a given *scale of observation*. Further, the changes of the system can be better defined in relation to variations in its status: from a normal (*i.e. *natural) to a pathological or altered state.
